# An Assessment of the Reliability and Factorial Validity of the Chinese Version of the Health Professional Education in Patient Safety Survey (H-PEPSS)

**DOI:** 10.3389/fpsyg.2019.02183

**Published:** 2019-09-25

**Authors:** Lingling Chen, Feifei Huang, Xiaohuan Yuan, Jihong Song, Linghui Chen

**Affiliations:** School of Nursing, Fujian Medical University, Fuzhou, China

**Keywords:** patient safety, nursing, reliability, item response theory, validity

## Abstract

**Background:**

A nursing student’s reflection on their knowledge and competence in patient safety (PS) may prepare them to provide safer care in certain circumstances. The Health Professional Education in PS Survey (H-PEPSS) is a validated tool for assessing the perceptions of nursing students with regards to competence in PS. The H-PEPSS is widely used internationally but is not available in Chinese.

**Objectives:**

This study aimed to translate the H-PEPSS into Chinese and test its psychometric properties among Chinese undergraduate nursing students.

**Design:**

This was a cross-sectional online survey that was conducted in 2018.

**Settings:**

Seven nursing schools in North, East, Northeast, Central, Southwest, South, and Northwest China.

**Participants:**

A total of 732 final-year undergraduate nursing students were recruited by convenience sampling.

**Methods:**

Translation was conducted rigorously in accordance with an adapted version of Brislin’s translation model. Psychometric evaluation was conducted by incorporating classical test theory and item response theory (IRT) analysis.

**Results:**

The Chinese version of the H-PEPSS (both the classroom and clinical practice versions) achieved a Cronbach’s α, marginal reliability and 2-week test-retest reliability of >0.85. A six-factor solution explaining 81.49% and 82.32% of the total variance was obtained for the classroom and clinical practice versions, respectively. This was further validated by confirmatory factor analysis. IRT analysis showed that the scale offers a broad range of information on PS competence and discriminates efficiently between patients with high and low levels of competence in PS.

**Conclusion:**

The Chinese version of the H-PEPSS is a reliable and valid instrument that is capable of evaluating competence in PS perceived by undergraduate nursing students. In addition, the survey may also be used to evaluate gaps in classroom knowledge and clinical competence, and to offer valid data for designing or tailoring new education strategies.

## Introduction

Patient safety (PS) is broadly defined as the prevention of unnecessary harm to patients and has become a serious and significant international public health issue (Usher et al., 2017). Globally, up to 10% of hospitalized patients experience some form of unintentional harm or adverse event that could have been prevented (World Health Organization [WHO], 2009). The world health organization (WHO) has reported that PS varies across different countries and is a particularly serious issue in developing countries (Long et al., 2011). In the Chinese context, as many as a quarter of hospital patient episodes of care involved at least one adverse event, with 40% being preventable; unfortunately, such events led to increased periods of hospitalization and even a risk of death (Zhu et al., 2013). Compared with other healthcare professionals, nurses tend to be in closer proximity to patients and are a constant presence at the bedsides of patients. As such, nurses have the potential to recognize, intercept and correct conditions that expose patients to risk at an early stage or even before harm occurs (Dubois et al., 2013; Hwang et al., 2016). Nursing students play a key role in nursing, but their PS-related knowledge and skills are insufficient (Sweeney et al., 2017); unfortunately, this lack of knowledge leads to a high prevalence of adverse effects among nursing students. It is estimated that approximately 40% of Chinese nursing students have made medical errors with patients, either directly or indirectly (Long et al., 2011; Ji, 2016).

It is important and necessary that healthcare institutions provide confident and safe care for patients, in line with best clinical practice and established standards (Usher et al., 2017). According to Bandura’s theory of self-efficacy, the confidence of medical students with regards to the knowledge and skills that are necessary for PS can influence both their behavior and performance in tasks (Bandura, 1988). It is therefore important to ensure that nursing students have confidence when learning about PS during their undergraduate education. Previous evidence indicated that nursing students were relatively confident in their learning ability with regards to the clinical dimensions of PS but were less confident about the sociocultural aspects of PS, such as working in teams and speaking up about PS issues (Ginsburg et al., 2013; Usher et al., 2017; VanDenKerkhof et al., 2017). However, these earlier lines of evidence focused only on developed countries (Ginsburg et al., 2013; Usher et al., 2017) and a few developing countries (e.g., Korea, Saudi Arabia, and Jordan) (Colet et al., 2015; Hwang et al., 2016; Suliman, 2019). In contrast, China has received only scant attention; while PS represents a particular concern in China, strategies and levels of education tends to lag behind those in developed countries (Liu et al., 2018). The clear shortfall of evidence with regards to how Chinese nursing students improve their knowledge and competence in PS may be due to a lack of valid tools or methods for evaluating PS competence in China, at least in part.

Evaluating the confidence of nursing students with regards to their confidence in PS is vital if we are to improve their future knowledge and competence. However, when evaluating PS, most previous studies have focused only on culture, attitude and knowledge; very few have addressed competence (Bressan et al., 2016). Most of the measurement instruments that are currently available evaluate safety reporting in the clinical setting (Cooper, 2013) or PS knowledge and attitudes aimed at improving student learning outcomes in the classroom (Christiansen et al., 2010). However, the psychometric analysis involved in most of these previous assessments was either exploratory or inadequate (Ginsburg et al., 2012; Bressan et al., 2016). In order to address this gap in knowledge, Ginsburg et al. (2012) developed the health professional education in patient safety survey (H-PEPSS) which aimed to specifically assess PS competence across a wide range of health professional groups (Ginsburg et al., 2012), including students.

The H-PEPSS may be used to assess PS knowledge learned from the classroom and PS competence acquired directly from clinical settings. In particular, the H-PEPSS can be used to investigate the gaps between classroom knowledge and clinical competence (Bressan et al., 2016; Hwang et al., 2016). Thus, the H-PEPSS is suitable for use by students who have recently completed, or have almost completed, their training, such as final-year nursing students (Ginsburg et al., 2012). The H-PEPSS has been translated into multiple languages and has been validated in many countries (Bressan et al., 2016; Hwang et al., 2016). For example, Bressan et al. (2016) validated the Italian version of the H-PEPSS in 574 undergraduate nursing students using explorative factor and reliability analysis. The Italian version of the H-PEPSS was shown to exhibit a Cronbach’s alpha of 0.94 in both the classroom and clinical practice versions; the six-factor structure was confirmed to explain 69.34 and 70.43% of the total variance of the scale for classroom and clinical practice, respectively. In another study, Hwang et al. (2016) translated the H-PEPSS into Korean and confirmed its reliability and validity; Cronbach’s alpha (a) was 0.91 across the entire scale and 0.70–0.81 for each of the six subscales. Unfortunately, however, there is no Chinese version of the H-PEPSS. It is possible that the H-PEPSS could be used to assess differences between classroom knowledge and clinical competence in the context of Chinese culture.

Another point to consider is that the existing H-PEPSS instruments from previous studies were developed or validated using the classical test theory (CTT), which is quantified based on the raw score across all items. In contrast, the item response theory (IRT) is a diverse family of models designed to represent the relationships between an individual’s item response and underlying latent traits (Hambleton et al., 1991). The characteristics of IRT could potentially offset the pitfalls of CTT by acquiring information relating to non-variant items, by analyzing latent items, by considering the standard errors of trait levels, and by analyzing rich items; collectively these analyses provide a much more robust evaluation (Hambleton et al., 1991). However, none of the previous studies have investigated the extent to which individual items may make a meaningful contribution to H-PEPSS scores. For example, it would be useful to be able to determine how well single items can discriminate (represented as a parameter in IRT) between individuals possessing high, moderate or low levels of specific measurable traits. Thus, the IRT analysis of scores derived from the H-PEPSS may provide stronger psychometric evidence and yield more accurate estimates of the parameters being tested.

In line with WHO recommendations, PS strategies in China are continuously being designed, tested and implemented in different clinical settings (Liu et al., 2018). For example, since 2007, the Chinese Hospital Association has issued an annual statement named “Ten goals for PS” (Chinese Hospital Management Association, 2014). Thus, efforts to strengthen the competency of nursing students in PS are urgently required in undergraduate healthcare curriculums. The premise of such efforts is to understand the extent of PS knowledge and competence among undergraduate nursing students (Doyle et al., 2015; Usher et al., 2017). Therefore, the purpose of the present study was to translate the H-PEPSS into Chinese and to investigate its psychometric properties among Chinese undergraduate nursing students by combining CTT and IRT analysis. We hypothesized that the combination of CTT and IRT would provide a powerful and robust means of assessing PS competence and knowledge when used with a Chinese version of the H-PEPSS.

## Materials and Methods

### Design

This was a cross-sectional study featuring several study sites. The study was approved by the ethical committee of Fujian Medical University (Reference: 2018054). All subjects voluntarily participated in the study and signed informed consent forms.

### Participants and Setting

This study used a convenience sampling method. To obtain a representative sample, we first selected one province for each of the seven Chinese administrative regions, which were representative of the population density, economic development and medical services of their respective regions. The seven administrative regions are as follows: North (Shanxi), East (Fujian), Northeast (Heilongjiang), Central (Hunan), Southwest (Guizhou), South (Hainan), and Northwest (Xinjiang) China. Secondly, in each selected province, we identified one provincial university that provided a baccalaureate nursing education program. According to Pett et al. (2003), 10–15 participants per item were considered to be appropriate for a target sample size. Assuming a non-response rate of 15%, the final appropriate sample size was identified as 770. Consequently, we invited all 110 final-year undergraduate nursing students at each university to participate. Finally, we received 732 valid survey responses, thus yielding an overall response rate of 95.06% across all universities.

One or two members of the nursing faculty at each participating university volunteered to be research partners and act as points of contact at their respective universities. The online questionnaire^1^ was made available to all eligible nursing students at each of the participating universities between September 2018 and January 2019. To encourage their participation, the students were told that this online survey was voluntary, anonymous and confidential. We also told them that 80% of participants would randomly receive a bonus (referred to as “Hongbao”in Chinese) of 10 yuan after they completed the questionnaire.

### The Instrument

#### Translation of the H-PEPSS Into the H-PEPSS-Chinese Version (CV)

We first sought written permission from the original author (Liane Ginsburg) and then translated the H-PEPSS into Chinese; we did this on the basis of an adapted version of Brislin’s translation model, as applied for cross-cultural translation. This involved forward-translation, back-translation, linguistic adaptation and a final pilot study (Jones et al., 2001). First, two bilingual Chinese doctoral candidate nursing graduates independently translated the H-PEPSS from English into Chinese. Then, a committee consisting of the two bilingual translators, two authors and one nursing education expert, proofread and agreed on a draft of the H-PEPSS (CV). Next, two native English speakers, who were nursing graduates with doctoral degrees (blinded to the H-PEPSS), back-translated the draft H-PEPSS-CV into English. Another committee (the two native English speakers and two of the authors) compared the back-translation with the original to identify any linguistic inaccuracies. Subsequently, a panel of five experts in nursing, clinical practice, and linguistics, assessed the cultural and semantic equivalency, and the translation validity index (TVI), of the H-PEPSS-CV. The TVI was adapted from the content validity index (CVI) described by Tang and Dixon (2002). The translational relevance of each of the 16 items of the questionnaire was graded on a 4-point scale (1 = “*totally different*” and 4 = “*equivalent*”). The item TVI (I-TVI) was calculated as the number of experts assigning a relevance rating of 3 or 4 divided by the total number of experts. The TVI of the total scale (S-TVI) represented the mean of the I-TVI for each item. After the experts reached a consensus on all items, a pilot study was conducted; this involved a convenience sample of 100 nursing undergraduate students at a nursing school in East China. These students evaluated the fluency, readability and comprehensibility of the items. We then made appropriate modifications according to the feedback we received. Subsequently, the H-PEPSS-CV was ready for validation.

#### H-PEPSS-CV

The H-PEPSS was developed to measure the self-reported competence of health professionals and students with regards to PS (Ginsburg et al., 2012). The H-PEPSS focuses on student learning with regards to specific PS content and is composed of 20 items representing seven factors, one covering clinical safety issues (four items) and the remaining six covering the six dimensions of the Safety Competencies Framework which featured communicating effectively (three items), working in a team with other health professionals (three items), managing safety risks (three items), understanding human and environmental factors (two items), recognizing, responding to and disclosing adverse events and close calls (two items) and the culture of safety (three items). Participants were asked to indicate their agreement with each item with regards to the content learned in the classroom and in the clinical setting; in other words, the scale has two versions. All items are rated using a 5-point Likert-scale (1 = “*strongly disagree*” to 5 = “*strongly agree*”). The reliability and validity of the H-PEPSS has been confirmed by a series of studies (Ginsburg et al., 2012; Bressan et al., 2016; Hwang et al., 2016).

In accordance with previous studies involving the validation of the H-PEPSS (Ginsburg et al., 2012; Bressan et al., 2016; Hwang et al., 2016), four of the items included in the survey focused on clinical aspects of safety (e.g., infection control and hand hygiene); this helped participants to distinguish between clinical and socio-cultural aspects of PS. Thus, the other 16 items in the scale that cover the six dimensions of the Safety Competencies Framework (for both the classroom and clinical training versions of the H-PEPSS-CV) formed the primary focus of our analysis.

#### Socio-Demographic and Nursing Experience Data

We also collated sociodemographic and nursing experience data from the undergraduate nursing students participating in the survey, including age, gender, prior experience of PS education, experience of adverse events and disclosing behavior.

### Statistical Analysis

Data analysis was conducted using SPSS version 17.0 and Mplus 6.1. Missing data (approximately 8%) were replaced using full information maximum likelihood and *p* < 0.05 was considered to be significant. The total sample was divided randomly into two subsamples using the “select case” function in SPSS with the sample size set to approximately 50%. These two subsamples were then used for exploratory factor analysis (EFA) and confirmatory factor analysis (CFA), respectively.

The factorial validity of the H-PEPSS-CV was assessed using EFA and CFA. Principal component factor analysis and varimax rotation were used for EFA. The number of factors extracted was determined based on the screen plot and used an eigenvalue >1.0, factor loading >0.4, percentage of explained variance and interpretability (Huang et al., 2017). Following EFA, we used CFA to examine the “best-fit” model of the scale using the maximum likelihood method. Goodness of fit was evaluated using absolute and relative indices (Huang et al., 2017), including normalized χ^2^ (χ^2/^df) between 1.0 and 3.0 (*p* > 0.05), root-mean-square error of approximation (<0.08), comparative fit index, Tucker-Lewis index and normalized fit index (>0.9).

Item response theory analysis was performed across the total sample using the marginal maximum likelihood method. Prior to the estimation of parameters, the IRT assumptions of unidimensionality and the local item independence for each subscale were checked using factor analysis (Hambleton et al., 1991; Stump et al., 2012). On the basis of Samejima’s graded response model (GRM) (Samejima, 1997), we calculated one discrimination parameter (“a”), four difficulty parameters (β_ik_) and test information function (TIF) values; next, we plotted item characteristic curves. In this study, “a” represented each item’s ability to discriminate between students with high and low levels of PS competence while β_ik_ indicated the difficulty in moving from a response in a lower category (k-1) to the next category (k) for item i. A large β_ik_ indicates that the step up to the next response option is more difficult and requires higher levels of PS competence. The item characteristic curves provided information about how the participants used these response categories (Samejima, 1997).

The content validity of the H-PEPSS-CV was validated by CVI; this was calculated based on the percentage of items that were rated 3 “*strong related*” or 4 “*very strongly related*” by six committee nursing education professionals.

Finally, to evaluate the reliability of the H-PEPSS-CV, we used Cronbach’s α, mean inter-item correlations (MIICs) and test-retest reliability using the CTT approach. The intraclass correlation coefficient (ICC) was also used to examine the stability of the H-PEPSS-CV over time by administering it after a 2-week interval to a convenience sample of 50 undergraduate nursing students (20.5 ± 8.12 years old; 20 males, 30 females). The reliability of IRT models was judged according to marginal reliability, the amount of information provided by the individual items and the entire scale.

## Results

### Sociodemographic Data

The mean age of the 732 nursing students was 21.56 ± 0.96 years; the majority of students were female (91.80%). Across the entire cohort, 79.70 and 78.70% of the students had prior experience of PS education and adverse events, respectively, while 20.30 and 21.30% of the students had no such experience. Furthermore, 94.30% of nursing students had experienced disclosing behavior while 5.60% of the students had not.

### CTT Validity Testing of the H-PEPSS-CV

#### Factorial Validity of the H-PEPSS-CV

**FIGURE 1:**
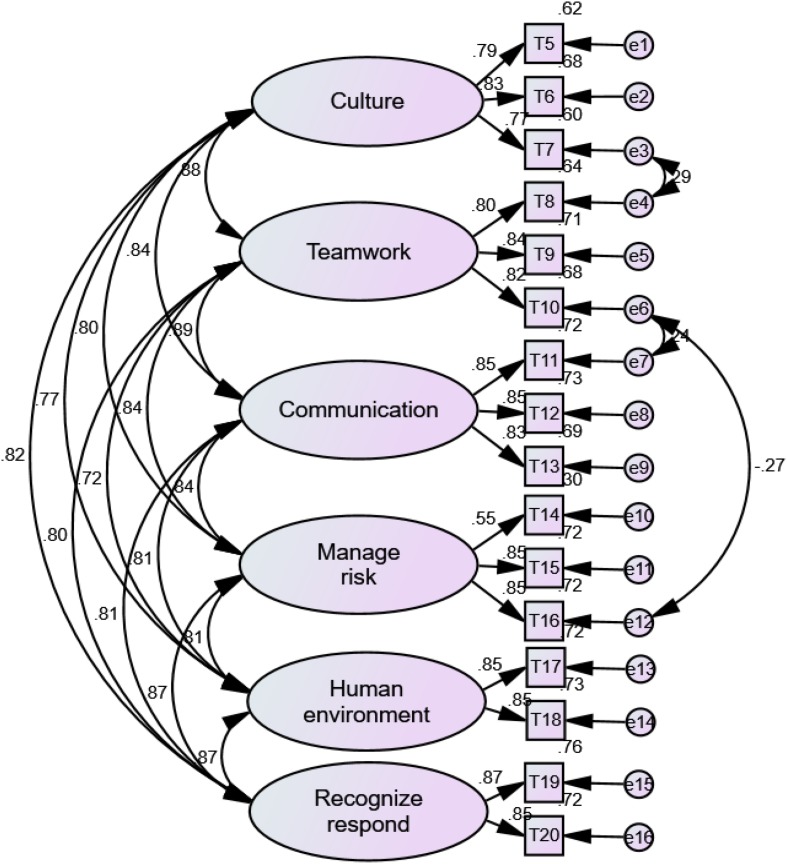
The factor structure of H-PEPSS-CV in classroom version *X*^2/df^ = 3.19 (*p* = 0.057), comparative fit index = 0.98, normed fit index = 0.97, Tucker-Lewis index = 0.97, and root-mean-square error of approximation = 0.055.

**FIGURE 2:**
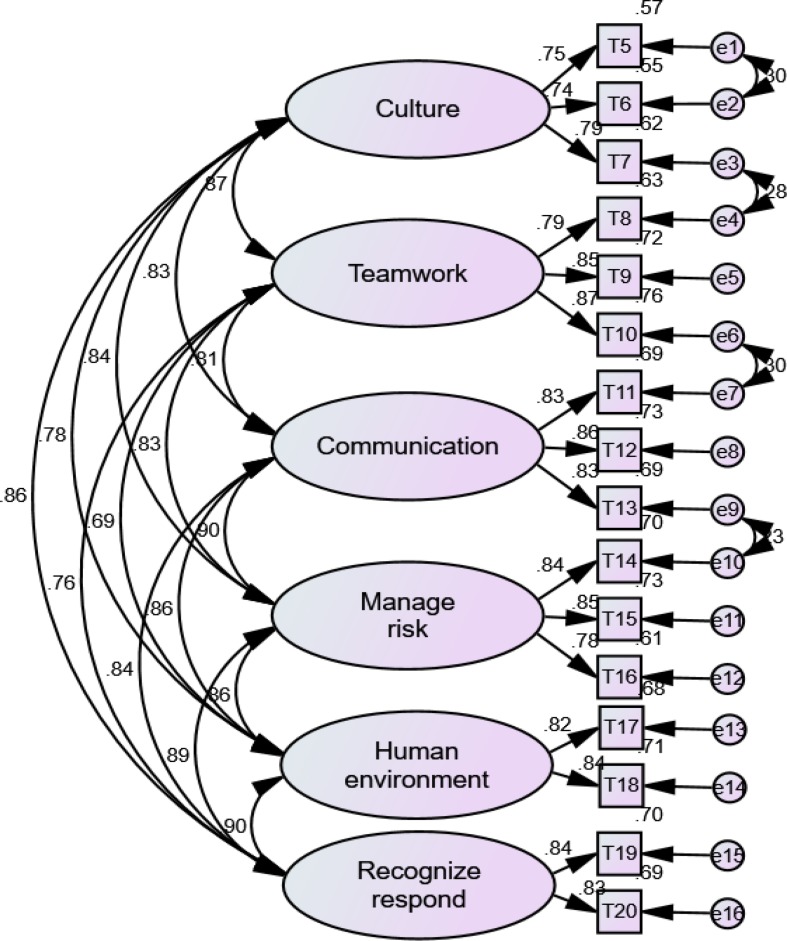
The factor structure of H-PEPSS-CV in clinical practice version*X*^2/df^ = 4.07 (*p* = 0.065), comparative fit index = 0.97, normed fit index = 0.96, Tucker-Lewis index = 0.97, and root-mean-square error of approximation = 0.065.

As shown in Tables 1, 2, the Kaiser-Meyer-Olkin (KMO) value and Bartlett’s test of sphericity for EFA were satisfied for both the classroom and clinical practice versions of the H-PEPSS-CV. A six-factor solution, explaining 81.49 and 82.32% of the total variance, was obtained for the classroom and clinical practice version, respectively. Factor loadings for all items of the classroom version were between 0.58 and 0.82 (*p* < 0.01), and between 0.47 and 0.80 (*p* < 0.01) for the clinical practice version. The six-factor structure was confirmed by CFA (Figures 1, 2), including safety culture (T5–T7), working in a team with health professionals (T8–T10), communicating effectively (T11–T13), managing safety risks (T14–T16), understanding human and environmental factors (T17,T18), and recognizing, responding to, and disclosing adverse events and close calls (T19,T20).

**TABLE 1:** Factor structure of the H-PEPSS-CV(Classroom version).

**Item**	**F1**	**F2**	**F3**	**F4**	**F5**	**F6**
T12. Enhancing patient safety through effective communication with other healthcare providers	0.85					
T11. Enhancing patient safety through clear and consistent communication with patients	0.85					
T13. Effective verbal and non-verbal communication abilities to prevent adverse events	0.83					
T18. The role of environmental factors such as work flow, ergonomics and resources, which effect patient safety		0.85				
T17. The role of human factors, such as fatigue, which effect patient safety		0.85				
T19. Recognizing an adverse event or close call			0.87			
T20. Reducing harm by addressing immediate risks for patients and others involved			0.85			
T9. Sharing authority, leadership and decision-making				0.84		
T8. Managing inter-professional conflict				0.80		
T10. Encouraging team members to speak up, question, challenge, advocate, and be accountable as appropriate to address safety issues				0.82		
T15. Identifying and implementing safety solutions					0.85	
T16. Anticipating and managing high risk situations					0.85	
T14. Recognizing routine situations in which safety problems may arise					0.55	
T6. The importance of a supportive environment that encourages patients and providers to speak up when they have safety concerns						0.83
T5. The importance of having a questioning attitude and speaking up when you see things that may be unsafe						0.79
T7. The nature of systems and system failures and their role in adverse events						0.77

Average variance extracted	0.71	0.72	0.74	0.67	0.58	0.64
Eigenvalue	2.85	2.85	2.37	1.97	1.88	1.12
Cumulative percentages	17.83	35.62	50.43	62.71	74.48	81.49

*H-PEPSS-CV: the Chinese version of the Health Professional Education in Patient Safety Survey. Kaiser-Meyer-Olkin test (0.959), Bartlett test was significant (χ^2^ = 8745.175, degrees of freedom = 120, *p* = 0.000).*

**TABLE 2:** Factor structure of the H-PEPSS-CV (Clinical practice version).

**Item**	**F1**	**F2**	**F3**	**F4**	**F5**	**F6**
T12	0.86					
T11	0.83					
T13	0.83					
T18		0.84				
T17		0.82				
T19			0.84			
T20			0.83			
T9				0.85		
T10			.	0.87		
T8			.	0.79		
T15					0.85	
T14					0.84	
T16					0.78	
T7						0.79
T6						0.74
T5						0.75

Average variance extracted.	0.71	0.69	0.70	0.70	0.68	0.59
Eigenvalue	2.45	1.84	1.86	2.65	2.30	2.08
Cumulative percentages	16.53	31.86	46.23	59.20	70.80	82.32

*H-PEPSS-CV: the Chinese version of the Health Professional Education in Patient Safety Survey. Kaiser-Meyer-Olkin test (0.959), Bartlett test was significant (χ^2^ = 8930.846, degrees of freedom = 120, *p* = 0.000).*

#### Content Validity of the H-PEPSS-CV

Expert consultation demonstrated that I-TVI and I-CVI ranged from 0.86 to 1.0 with an S-TVI and S-CVI of 0.89 and 0.87, respectively.

### IRT Analysis of the H-PEPSS-CV

The mean adjusted chi-square index was 1.5, thus supporting the use of GRM. The percentage of variance accounting for the first factor ranged from 28.91% to 40.42%, and the eigenvalue of the first factor divided by the second factor ranged from 3.34 to 4.56. These results were also confirmed by CFA (data not shown). Collectively, these test confirmed the unidimensionality of each of the six subscales. The parameter estimates (a, β_1_, β_2_, β_3_, and β_4_) in the IRT analysis for the 16 items are presented in Table 3. All items, for both classroom and clinical practice versions, showed high discriminatory ability for measuring PS competence, with an “a” ≥1 (Hambleton et al., 1991). For the classroom version, the β_ik_ (for all items) was between –2.83 (I17, I18) and 1.68 (I16), with no disordinal items or reversals. For the clinical practice version, β_ik_ ranged from –2.79 (I5) to 1.47 (I8), with no disordinal items or reversals. These results indicated that the items offer a broad range of information and low difficulty for nursing students.

**TABLE 3:** Item response theory parameter estimates for the H-PEPSS-CV.

**Item**	**Classroom version**							**Clinical practice version**						
		
	**Slope**	**Difficulty**				**Maximum TIF value**	**Mean TIF^∗^ value**	**Slope**	**Difficulty**				**Maximum TIF value**	**Mean TIF^∗^ value**
								
	**α (SE)**	**β1 (SE)**	**β2 (SE)**	**β3 (SE)**	**β4 (SE)**			**α (SE)**	**β1 (SE)**	**β2 (SE)**	**β3 (SE)**	**β4 (SE)**		
I5	2.32 (0.16)	−2.61 (0.33)	−1.93 (0.17)	−0.58 (0.07)	1.25 (0.08)	1.60	1.02	2.49 (0.18)	−2.79 (0.41)	−2.26 (0.29)	−0.89 (0.09)	0.86 (0.07)	1.84	1.09
I6	2.50 (0.16)	−2.35 (0.26)	−1.59 (0.13)	−0.45 (0.07)	1.28 (0.08)	1.82	1.12	2.32 (0.17)	−2.72 (0.34)	−1.91 (0.20)	−0.66 (0.08)	1.02 (0.08)	1.58	1.02
I7	2.60 (0.16)	−2.62 (0.32)	−1.55 (0.12)	−0.24 (0.06)	1.36 (0.08)	1.83	1.24	2.65 (0.17)	−2.47 (0.30)	−1.88 (0.18)	−0.45 (0.06)	1.15 (0.07)	2.08	1.20
I8	2.60 (0.17)	−2.55 (0.34)	−1.41 (0.11)	−0.08 (0.05)	1.66 (0.09)	1.78	1.27	2.39 (0.16)	−2.46 (0.33)	−1.66 (0.16)	−0.18 (0.06)	1.47 (0.09)	1.66	1.09
I9	2.63 (0.16)	−2.56 (0.28)	−1.53 (0.12)	−0.09 (0.06)	1.57 (0.09)	1.87	1.28	2.55 (0.18)	−2.44 (0.39)	−1.64 (0.15)	−0.33 (0.06)	1.42 (0.09)	1.86	1.17
I10	2.85 (0.19)	−2.58 (0.29)	−1.61 (0.13)	−0.39 (0.06)	1.43 (0.09)	2.19	1.38	3.12 (0.24)	−2.13 (0.31)	−1.63 (0.17)	−0.45 (0.06)	1.21 (0.07)	2.89	1.44
I11	3.47 (0.23)	−2.29 (0.26)	−1.47 (0.10)	−0.43 (0.05)	1.34 (0.07)	3.21	1.66	3.31 (0.24)	−2.42 (0.53)	−1.87 (0.18)	−0.66 (0.06)	0.97 (0.06)	3.20	1.59
I12	3.10 (0.20)	−2.30 (0.25)	−1.50 (0.11)	−0.39 (0.06)	1.40 (0.08)	1.79	1.46	3.34 (0.25)	−2.38 (0.32)	−1.97 (0.23)	−0.67 (0.06)	0.99 (0.06)	3.20	1.58
I13	3.29 (0.19)	−2.28 (0.26)	−1.50 (0.11)	−0.33 (0.05)	1.26 (0.07)	2.96	1.46	3.32 (0.24)	−2.14 (0.33)	−1.70 (0.17)	−0.60 (0.06)	1.00 (0.06)	3.25	1.54
I14	3.42 (0.22)	−2.42 (0.37)	−1.57 (0.12)	−0.36 (0.05)	1.38 (0.08)	3.11	1.65	3.56 (0.28)	−2.29 (0.61)	−1.87 (0.22)	−0.61 (0.06)	1.09 (0.06)	3.73	1.70
I15	3.00 (0.18)	−2.40 (0.33)	−1.54 (0.12)	−0.23 (0.05)	1.48 (0.08)	2.45	1.46	3.46 (0.26)	−2.32 (0.57)	−1.62 (0.14)	−0.38 (0.05)	1.20 (0.06)	3.28	1.68
I16	2.77 (0.16)	−2.21 (0.23)	−1.40 (0.11)	0.05 (0.05)	1.68 (0.09)	2.15	1.35	2.67 (0.19)	−2.36 (0.29)	−1.51 (0.12)	−0.08 (0.06)	1.42 (0.09)	1.99	1.27
I17	2.66 (0.18)	−2.83 (0.64)	−1.76 (0.15)	−0.54 (0.06)	1.42 (0.09)	1.92	1.33	2.69 (0.20)	−2.67 (0.46)	−1.89 (0.20)	−0.76 (0.08)	1.02 (0.07)	2.07	1.26
I18	2.69 (0.17)	−2.83 (0.63)	−1.65 (0.13)	−0.45 (0.06)	1.47 (0.09)	1.94	1.31	2.95 (0.19)	−2.69 (0.51)	−1.85 (0.20)	−0.64 (0.06)	1.10 (0.07)	2.40	1.45
I19	3.09 (0.19)	−2.28 (0.25)	−1.52 (0.13)	−0.32 (0.05)	1.47 (0.08)	2.64	1.47	3.00 (0.21)	−2.70 (0.37)	−1.96 (0.22)	−0.58 (0.06)	1.14 (0.07)	2.53	1.48
I20	3.04 (0.19)	−2.32 (0.26)	−1.62 (0.12)	−0.39 (0.05)	1.51 (0.09)	2.62	1.43	3.06 (0.21)	−2.68 (0.34)	−1.87 (0.18)	−0.62 (0.07)	1.22 (0.07)	2.58	1.51

*IIF, item information function. ^∗^The mean item information function of seven categories (category: −3, −2, −1, 0, 1, 2, 3).*

The maximum information function value for the classroom and clinical practice versions ranged from 1.60 (I5) to 3.21 (I12), and from 1.58 (I6) to 3.72 (I14), respectively. The mean information function value for each item was >1. For both versions of the H-PEPSS-CV, the item characteristic curves (shown in Supplementary Appendices A,B) had appropriate shapes, with the peak of the five curves not overlapping and curves 2, 3, and 4 being normally distributed. Regarding TIF, both versions of H-PEPSS-CV gathered information most precisely when θ ranged from −2.0 to −2.8.

### Reliability of the H-PEPSS-CV

For the classroom version of the H-PEPSS-CV, Cronbach’s α, MIIC, marginal reliability and ICC for the total scale were 0.95, 0.55, 0.96, and 0.88, respectively. For clinical practice, Cronbach’s α, MIIC, marginal reliability and ICC for the total scale were 0.96, 0.58, 0.96, and 0.87, respectively. These findings indicate that H-PEPSS-CV achieved satisfactory reliability in terms of Cronbach’s α, ICC and with a marginal reliability ≥0.7 and an MIIC ≥0.3 (Zhao, 2014). In addition, the classroom and clinical practice versions of the H-PEPSS-CV provided the most precise information, with the lowest standard error, when participants had estimated PS competence levels ranging from −2.5 to −1.5, and from 1 to 1.5, respectively (Supplementary Appendices C,D).

### Differences in Scores Between the Classroom and Clinical Practice Version of the H-PEPSS-CV

There was a statistically significant difference between the scores for PS learning in the classroom and clinical practice (*t* = 2.61, *p* = 0.00). At the dimension level, classroom learning increased confidence in “clinical safety skills,” “working in teams with other health professionals,” and “communicating effectively” to a greater extent than learning in the clinical setting (*t* = 6.84, 9.34, 7.41, *p* = 0.00). The mean scores of PS dimensions for “understanding human and environmental factors” and “managing safety risks” were significantly higher in the clinical setting compared with the classroom (*t* = 8.54, 5.95, *p* = 0.00).

## Discussion

### General Discussion

Previous research reported a high rate (40–53.2%) of preventable adverse events caused by Chinese undergraduate nursing students. This is a cause for concern given that PS is emerging as a significant priority in China. Helping students to reflect on their knowledge and competence in PS may help prepare them to provide safer care in a variety of different circumstances (Stevanin et al., 2015). However, to the best of our knowledge, no tools have been designed or validated for the specific evaluation of PS knowledge and competence in the context of Chinese nursing education. Although there is a lack of consensus with regards to the best tool to adopt, the H-PEPSS was developed in accordance with the Canadian PS Institute’s Safety Competencies Framework and has been widely used internationally with good psychometric testing results (Ginsburg et al., 2012). The present study is the first to examine the application of the H-PEPSS in China. Translation was conducted rigorously to ensure that equivalence was established. Our psychometric evaluation also showed that the H-PEPSS-CV is a reliable and valid instrument for assessing the self-reported PS knowledge and competence of Chinese nursing students.

Notably, the existing H-PEPSS instruments were developed and validated by focusing on the conventional CTT approach (Ginsburg et al., 2012; Bressan et al., 2016; Hwang et al., 2016); in this approach, the respondent characteristic of interest was quantified based on the raw score across all items. In contrast, IRT analysis provides a sophisticated index of measurement precision, representing how precisely/reliably a specific item/scale contributes to the measurement of the latent trait at different levels (Hambleton et al., 1991). The present study takes a rare approach in that the H-PEPSS-CV was evaluated using both CTT and IRT approaches.

Similar to the cross-validation of H-PEPSS in different cultures (Bressan et al., 2016; Hwang et al., 2016), our EFA and CFA results support the fact that the H-PEPSS-CV shares the same six-factor structural model with the original tool (Ginsburg et al., 2012), which therefore includes the stable concept framework set by Ginsburg and colleges, but also confirms adequate factorial validity of the scale. Consistent with the original scale, the Chinese version of the H-PEPSS has two versions, one for measuring PS knowledge developed in the classroom and one for measuring competence developed during clinical training. The explained variance for both versions was >60% and the eigenvalue was >1 for each singular factor, thus demonstrating the ability of the scale to evaluate PS knowledge and competence as perceived by nursing students (Bressan et al., 2016). Furthermore, the H-PEPSS-CV showed satisfactory internal consistency and good temporal stability. IRT analysis also confirmed that the H-PEPSS-CV was supported by marginal reliability and that information function values were obtained for both individual items and the overall scale.

Our IRT analysis provided useful findings relating to the function of items and information relating to the test scales. Our data further indicated that the H-PEPSS-CV was able to differentiate between nursing students with varying levels of PS knowledge. When represented graphically, high TIF values were associated with low standard errors of measurement, thus indicating precision (Hambleton et al., 1991). We found that either the classroom version or the clinical practice version of the H-PEPSS-CV could precisely and reliably measure PS competence in nursing students, even in those with low levels of competence. In short, these findings provide good evidence that the H-PEPSS-CV is highly useful for both practice and research in nursing education.

In accordance with earlier studies (Ginsburg et al., 2013; Usher et al., 2017; Suliman, 2019), we also observed a difference in the confidence of nursing students when comparing their learning in the classroom and in clinical settings. This further highlights the gap between nursing education and clinical practice (Ginsburg et al., 2013; Usher et al., 2017). However, our data indicate that the H-PEPSS-CV is likely to be very useful for evaluating gaps between classroom knowledge and clinical competence.

### Limitations and Future Directions for Research

This study has some limitations that should be considered. First, although students were recruited from a number of centers in China, the convenience sampling method may have led to sample bias and may have affected the generalizability of our findings. Second, we relied solely on self-reported data; this may have led to social desirability bias. It is therefore necessary to analyze the sensitivity and specificity of our new tool for predicting the actual lack of PS knowledge and competence in students, as evaluated by formal examinations, including objective and structured clinical examinations. Third, we did not investigate whether sensitivity changed over time. It is now necessary to determine whether the H-PEPSS-CV is applicable for the longitudinal monitoring and evaluation of the efficacy of nursing education programs. Fourth, the H-PEPSS features two factors which only contain two items, thus potentially affecting the stability of these two factors. Further research is required to investigate this possibility. Finally, we now need to acquire additional evidence of validity, such as convergent and discriminant validity; we also need to assess measurement invariance of the scale. In future studies, it is important to carry out correlation analysis of H-PEPSS-CV with other scales which measure similar or different constructs, and to use differential item function analysis to confirm that items function in a similar way across different groups and different cultures.

### Implications for Research and Practice

Patient safety is critical for the provision of quality healthcare and should be a central component of undergraduate nursing education. In this context, the H-PEPSS is widely recommended as a reliable and valid instrument with which to measure the perceptions of students and their competence in PS (Stevanin et al., 2015). The H-PEPSS-CV can be used to by nursing students to self-assess their knowledge and competence of PS. This may contribute to increased levels of student awareness during safety education. For nursing educators, the H-PEPSS-CV is useful for analyzing gaps between theoretical knowledge in the classroom and the competence developed from clinical practice. It also provides an important tool for designing and tailoring specific educational strategies and for the evaluation of their effectiveness. For nursing managers, the H-PEPSS-CV may also be useful for evaluating the quality of clinical placements attended by nursing students, and may contribute to the improvement of first-year training programs for newly graduated nurses. Furthermore, the H-PEPSS-CV also creates a basis for local, national, and international comparisons relating to self-reported PS knowledge and competence.

## Conclusion

The H-PEPSS-CV is a 16-item, self-reported, six-dimensional instrument that can be used to assess the PS competence perceived by undergraduate students in nursing and other healthcare disciplines. This instrument may help students to self-assess their competence with regards to PS issues. The H-PEPSS-CV may also be useful for evaluating gaps in classroom knowledge and clinical competence, thereby providing valid data for designing or tailoring new education strategies.

## Data Availability Statement

The datasets generated for this study are available on request to the corresponding author.

## Ethics Statement

The studies involving human participants were reviewed and approved by the ethical committee of Fujian Medical University (2018054). The patients/participants provided their written informed consent to participate in this study.

## Author Contributions

FH contributed to the conception and design of the work. LlC contributed to the interpretation of data and drafting of the manuscript. XY contributed to the data collection and analysis. JS and LhC contributed to the translation of instruments. All authors contributed to the approval of the final manuscript for publication.

## Conflict of Interest

The authors declare that the research was conducted in the absence of any commercial or financial relationships that could be construed as a potential conflict of interest.

## Footnotes

1 Hosted by Wenjuanxing, http://www.wjx.cn, a popular online survey platform in China.
