# Supplementary material for: An Assessment of the Reliability and Factorial Validity of the Chinese Version of the Health Professional Education in Patient Safety Survey (H-PEPSS)
Source: Front Psychol. 2019 Sep 25;10:2183. doi: 10.3389/fpsyg.2019.02183 (PMC6774353; doi:10.3389/fpsyg.2019.02183)
Supplement: Supplementary file 1 [file Data_Sheet_1.docx]

Appendix A

Appendix A. Illustration of item characteristic curves, and test information function for all items in the classroom.

*Note.* Curve 1,2, 3,4 and 5 represents the probability of a patient choosing each of the response options, 1 (not at all), 2 (a bit), 3 (moderately), 4 (strongly) and 5 (very strongly), respectively.

Appendix B

Appendix C

Appendix B. Illustration of test information function and standard error curves for the Leiden index of depression sensitivity-modified Chinese version-Classroom

Appendix B. Illustration of item characteristic curves, and test information function for all items in the clinical practice.

*Note.* Curve 1,2, 3,4 and 5 represents the probability of a patient choosing each of the response options, 1 (not at all), 2 (a bit), 3 (moderately), 4 (strongly) and 5 (very strongly), respectively.

Appendix C

Communicating effectively（* at -2.5θ） Culture of safety（* at -2.5θ）

Managing safety risks（* at -2.4θ） Recognizing, responding to, and disclosing adverse events and close calls（* at -2.5θ）

Understanding human and environmental Working in a team with their health

factors（* at -2.0θ） professionals（* at -2.4θ）

*

*

*

*

*

*

Appendix C. Illustration of test information function and standard error curves for the Chinese version of the Health professional education in patient safety survey-Classroom version

Note. Solid lines; left y-axis=total information aggregated across all items assessing patient safety competence in the clinical practice along the latent trait (θ, theta) ranging from −3 to 3. Dashed lines; right y-axis=standard error of measurement for it along the latent trait (θ, theta) ranging from −3 to 3.

*Most precise information gathered.

Appendix D

Communicating effectively（* at -2.5θ） Culture of safety（* at -2.6θ）

Managing safety risks（* at -2.5θ） Recognizing, responding to, and disclosing adverse events and close calls（* at -2.8θ）

Understanding human and environmental Working in a team with their health

factors（* at -2.5θ） professionals（* at -2.4θ）

*

*

*

*

*

*

Appendix D. Illustration of test information function and standard error curves for the Chinese version of the Health professional education in patient safety survey-Clinical practice version

Note. Solid lines; left y-axis=total information aggregated across all items assessing patient safety competence in the clinical practice along the latent trait (θ, theta) ranging from −3 to 3. Dashed lines; right y-axis=standard error of measurement for it along the latent trait (θ, theta) ranging from −3 to 3.

*Most precise information gathered.
